# The genetic effects of hormones modulated by the Pituitary-Thyroid/Adrenal/Gonadal axis on the risk of developing venous thromboembolism: a mendelian randomization study

**DOI:** 10.1186/s12872-024-04039-y

**Published:** 2024-07-25

**Authors:** Hao Tian, Chaozheng Xie, Biyun Teng, Qiu Zeng, Yu Zhao, Fenghe Li, Chuli Jiang, Zheng Chen

**Affiliations:** 1 Department of Vascular Surgery, The First Affiliated Hospital of Chongqing Medical University, 1 N. Youyi Street, Chongqing, 400016 China; 2 Department of Gastrointestinal Surgery, The First Affiliated Hospital of Chongqing Medical University, Chongqing, China

**Keywords:** Venous thromboembolism, Pituitary hormones, Thyroid hormones, Adrenal cortex hormones, Gonadal hormones, Mendelian randomization analysis

## Abstract

**Background:**

The aim of this study was to explore the genetic effects of hormones modulated through the pituitary-thyroid/adrenal/gonadal axis on the risk of developing venous thromboembolism (VTE) and to investigate the potentially causal relationships between them.

**Methods:**

A two-sample Mendelian randomization (MR) design was used. The single-nucleotide polymorphisms (SNPs) used as instrumental variables for various hormones and hormone-mediated diseases were derived from published genome-wide association studies (GWASs). Summary statistics for the risk of developing VTE (including deep venous thrombosis [DVT] and pulmonary embolism [PE]) were obtained from the UK Biobank and the FinnGen consortium. Inverse-variance weighting (IVW) was applied as the primary method to analyse causal associations. Other MR methods were used for supplementary estimates and sensitivity analysis.

**Results:**

A genetic predisposition to greater free thyroxine (FT4) concentrations was associated with a greater risk of developing DVT (OR = 1.0007, 95%CI [1.0001–1.0013], *p* = 0.0174) and VTE (OR = 1.0008, 95%CI [1.0002–1.0013], *p* = 0.0123). Genetically predicted hyperthyroidism was significantly associated with an increased risk of developing DVT (OR = 1.0685, 95%CI [1.0139–1.1261], *p* = 0.0134) and VTE (OR = 1.0740, 95%CI [1.0165–1.1348], *p* = 0.0110). According to the initial MR analysis, testosterone concentrations were positively associated with the risk of developing VTE (OR = 1.0038, 95%CI [1.004–1.0072], *p* = 0.0285). After sex stratification, estradiol concentrations were positively associated with the risk of developing DVT (OR = 1.0143, 95%CI [1.0020–1.0267], *p* = 0.0226) and VTE (OR = 1.0156, 95%CI [1.0029–1.0285], *p* = 0.0158) in females, while the significant relationship between testosterone and VTE did not persist. SHBG rs858518 was identified as the only SNP that was associated with an increased risk of developing VTE, mediated by estradiol, in females.

**Conclusions:**

Genetically predicted hyperthyroidism and increased FT4 concentrations were positively associated with the risk of developing VTE. The effects of genetically predicted sex hormones on the risk of developing VTE differed between males and females. Greater genetically predicted estradiol concentrations were associated with an increased risk of developing VTE in females, while the SHBG rs858518 variant may become a potential prevention and treatment target for female VTE.

**Supplementary Information:**

The online version contains supplementary material available at 10.1186/s12872-024-04039-y.

## Background

Venous thromboembolism (VTE), including deep vein thrombosis (DVT) and pulmonary embolism (PE), is the most common cardiovascular disease following myocardial infarction and stroke, with high life-threatening risks [1, 2].

The pathogenesis of VTE involves blood hypercoagulability, vascular damage, and flow alterations [1–3]. Disorders of hormones modulated through the pituitary-target organ axis and subsequent endocrine diseases have been reported to contribute to abnormal coagulation function and fibrinolytic system activity. They are, therefore, considered risk factors for VTE [4, 5]. For example, observational studies indicated that patients diagnosed with hyperthyroidism (or with elevated free thyroxine [FT4]) and hypercortisolism (e.g., Cushing’s syndrome) were associated with increased coagulation factors (e.g., FVIII, FIX, FX) and von Willebrand factor (VWF), leading to a greater risk of developing VTE [2, 5–8]. Elevated sex hormones (e.g., estrogen and progesterone) are also associated with an increased risk of developing VTE in women [9, 10]. However, these findings remain controversial because observational studies are prone to biases in study design, residual confounding, and reverse causality [11, 12]. The observed associations between hormones and VTE cannot be considered causal relationships [13].

Mendelian randomization (MR) studies mimic randomized clinical trials (RCTs) by leveraging allelic randomization during meiosis and subsequent irreversible exposure to genotype at conception. In MR analysis, researchers use genetic variants, usually single-nucleotide polymorphisms (SNPs), as instrumental variables (IVs) to substitute for risk factors and then calculate their statistical influence on diseases or outcomes. Differences in SNPs data can stratify a specific population into different subsets, similar to treated groups and control groups in RCTs [14]. It is therefore less likely to be affected by environmental factors, social confounding factors and reverse causality, compared with traditional observational studies [13, 15, 16]. In addition, two-sample MR studies that combine information from two separate genome-wide association studies (GWASs) can offer proofs for causality with less disputation due to the large sample size and enhanced statistical power [17].

Therefore, we performed a large-scale, systematic two-sample MR study of the association between hormones modulated through pituitary-thyroid/adrenal/gonadal axis and VTE (including DVT and PE), aiming to explore the genetic effects of these hormones on the risk of developing VTE and to identify the potentially causality of those relationships.

## Methods

### Study design

**Fig. 1:**
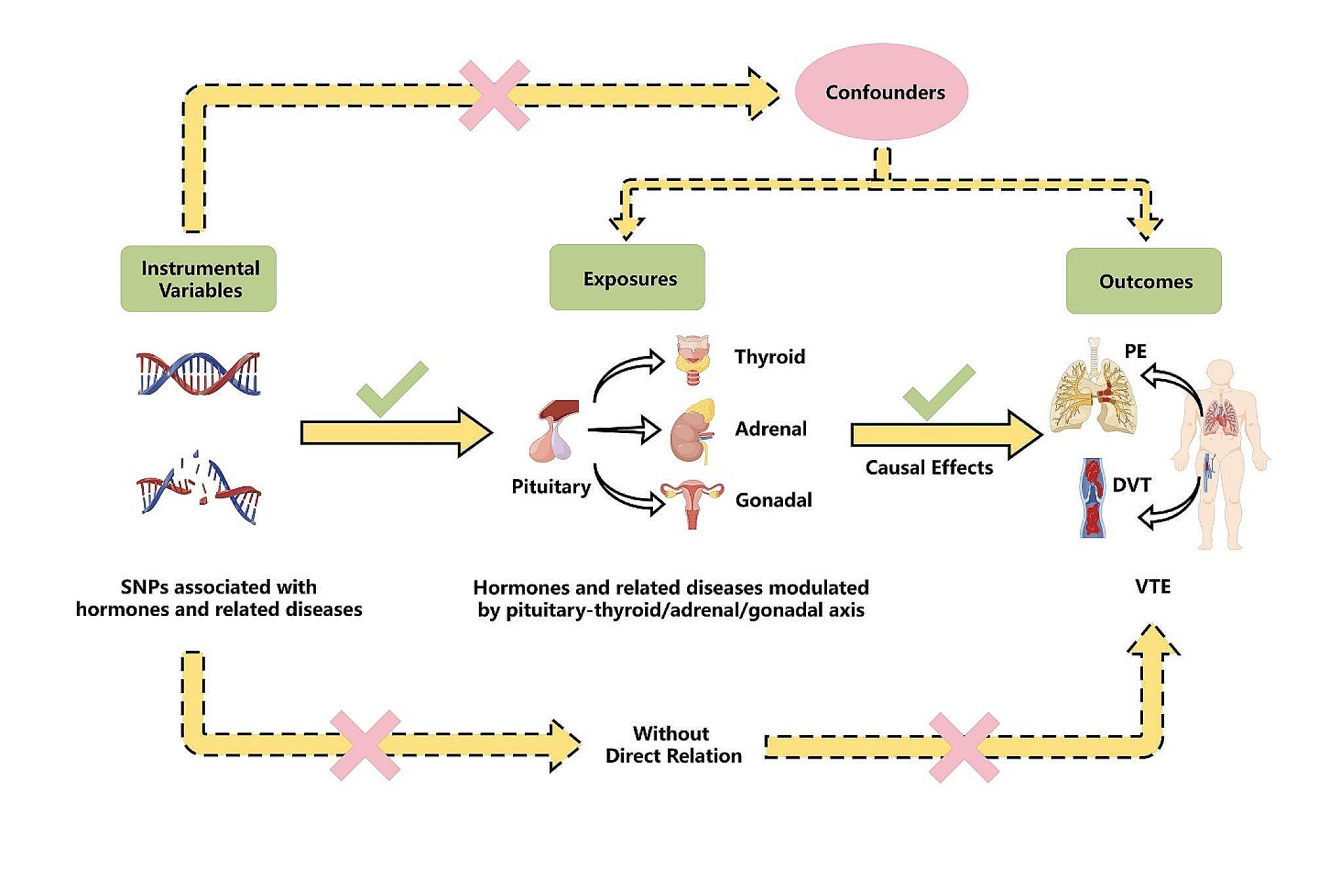
Schematic view and three assumptions of this Mendelian Randomization study. (By Figdraw) SNPs, single-nucleotide polymorphisms; VTE, venous thromboembolism; DVT, deep vein thrombosis; PE, pulmonary embolism

A schematic view of the study and the three MR assumptions are shown in Fig. 1. The genetic variants associated with various hormones and hormone-mediated diseases modulated by the pituitary-thyroid/adrenal/gonadal axis were used as genetic IVs to examine the causal associations between those IVs and the risk of developing VTE, DVT and PE. Three key assumptions need to be met to conduct an MR study. First, the selected genetic variants should be strongly correlated with exposure. Second, the genetic variants must be independent of potential confounding factors. Finally, the influence of genetic variants on outcomes should only be mediated through exposure [18]. Since the expression of sex hormones has sex-specific differences, sex stratification analysis for the effect of genetically predicted estradiol and testosterone on the risk of developing VTE was further conducted. Written informed consent was obtained from the participants for all the included GWASs, and the original studies were all approved by the appropriate ethics committees. As we only extracted data from the GWASs database for analysis, no additional ethical review was required for this study.

### Data sources and SNPs selection

The genetic IVs for various hormones and hormone-mediated diseases were derived from published GWASs. Summary statistics for the risk of developing VTE, DVT and PE were obtained from the UK Biobank (UKB) (http://www.nealelab.is/uk-biobank) and FinnGen (www.finngen.fi/en/). The details regarding the data sources are presented in Supplementary Table 1. In the sex stratification analysis, we used sex-specific summary statistics for genetically predicted sex hormones and the risk of developing DVT, PE and VTE, which were derived from the UKB.

SNPs were selected as IVs if they were associated with the exposure at the genome-wide significance level (< 5 × 10^− 8^). For some exposures without enough SNPs selected, a less conservative threshold (*P* < 1 × 10^− 5^) was applied to avoid missing positive results. Moreover, independent SNPs (r^2^ < 0.001, window size of 10,000 kb) were selected as IVs to avoid offsets caused by linkage disequilibrium. The F-statistics of most of these SNPs were above the threshold of 10, indicating that they strongly represent hormones and hormone-mediated diseases included in this MR analysis.

### Statistical methods

The inverse-variance weighting (IVW) method was used as the primary method for assessing the causal associations between the exposures and outcomes, supplemented by a variety of analyses, such as MR-Egger, weighted median, simple mode, Cochrane’s Q test, and MR-PRESSO (MR Pleiotropy RESidual Sum and Outlier) [19–22]. For MR analyses with only one IV, the Wald ratio was used instead of the IVW to evaluate the causal relation accurately. Directional pleiotropy was assessed and corrected by MR-Egger regression model analysis, in which intercepts with *p* values < 0.05 were considered to indicate the presence of horizontal pleiotropy. The simple mode method provided robustness for pleiotropy. The weighted median method gives consistent causal estimates even when half the weight in the MR analysis comes from invalid IVs. Cochrane’s Q test was performed to assess the heterogeneity of selected SNPs. In addition, the potential outliers detected by the MR-PRESSO method were replaced.

All the statistical results were two-sided, and *P* < 0.05 was considered to indicate statistical significance. The statistical analyses were performed using the “Two-Sample-MR” and “MR-PRESSO” packages in R software (Version 4.3.1).

## Results

**Fig. 2:**
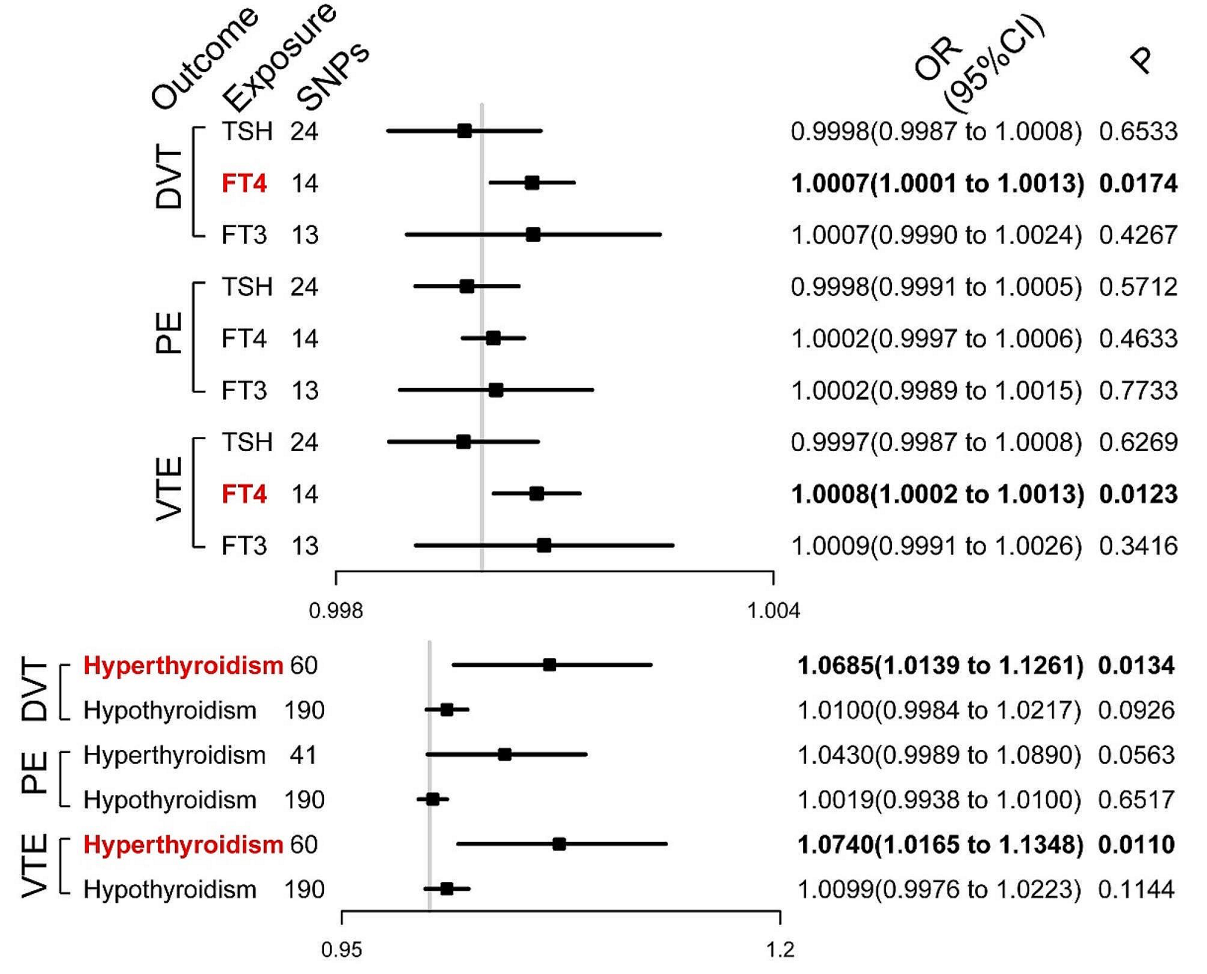
Effects of hormones and related diseases modulated by the pituitary-thyroid axis on the risk of venous thromboembolism (VTE), deep vein thrombosis (DVT), and pulmonary embolism (PE). SNPs, single-nucleotide polymorphisms; OR, odds ratio; CI, confidence interval; TSH, thyroid stimulating hormone; FT4, free thyroxine; FT3, free triiodothyronine. Bold values indicate the statistical significance at a level of *P* < 0.05

As shown in Fig. 2, genetic predisposition to greater FT4 concentrations was associated with a greater risk of developing DVT and VTE, with odds ratios (ORs) of 1.0007 (95%CI [1.0001–1.0013], *P* = 0.0174) and 1.0008 (95%CI [1.0002–1.0013], *P* = 0.0123), respectively. Genetically predicted hyperthyroidism was associated with a greater risk of DVT (OR = 1.0685, 95%CI [1.0139–1.1261], *P* = 0.0134) and VTE (OR = 1.0740, 95%CI [1.0165–1.1348], *P* = 0.0110). However, the effects of elevated FT4 concentrations and hyperthyroidism status on the risk of developing PE were not significant. In addition, thyroid stimulating hormone (TSH) concentrations, free triiodothyronine (FT3) concentrations, and hypothyroidism status had no significant causal associations with the risk of developing DVT, PE or VTE.

**Fig. 3:**
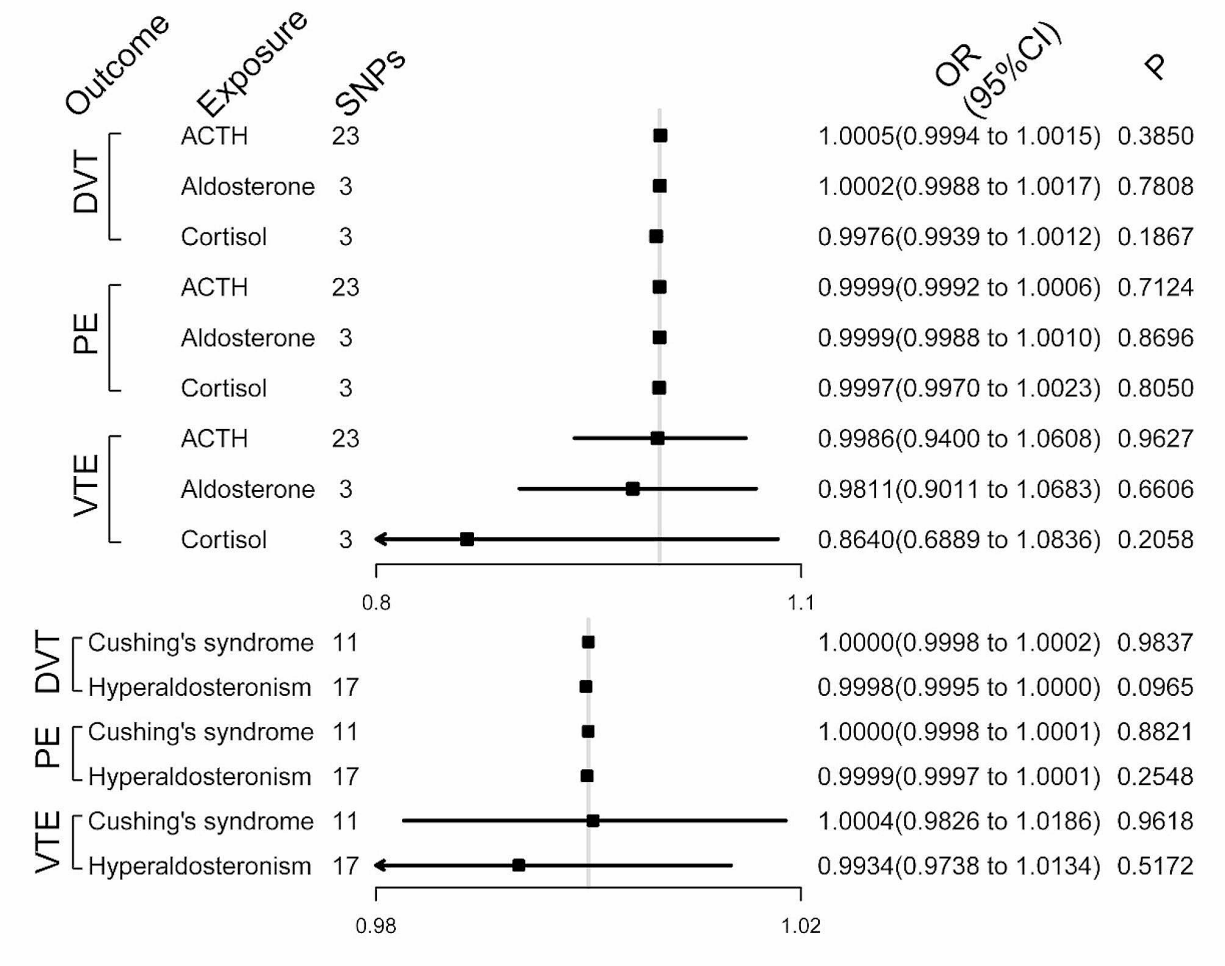
Effects of hormones and related diseases modulated by the pituitary-adrenal axis on the risk of venous thromboembolism (VTE), deep vein thrombosis (DVT), and pulmonary embolism (PE). SNPs, single-nucleotide polymorphisms; OR, odds ratio; CI, confidence interval; ACTH, adrenocorticotropic hormone. Bold values indicate the statistical significance at a level of *P* < 0.05

The included hormones regulated by the pituitary-adrenal axis (i.e., adrenocorticotropic hormone [ACTH], aldosterone and cortisol) and related diseases (e.g., Cushing’s syndrome and hyperaldosteronism) had no significant causal relationships with the risk of developing DVT, PE, or VTE (Fig. 3).

**Fig. 4:**
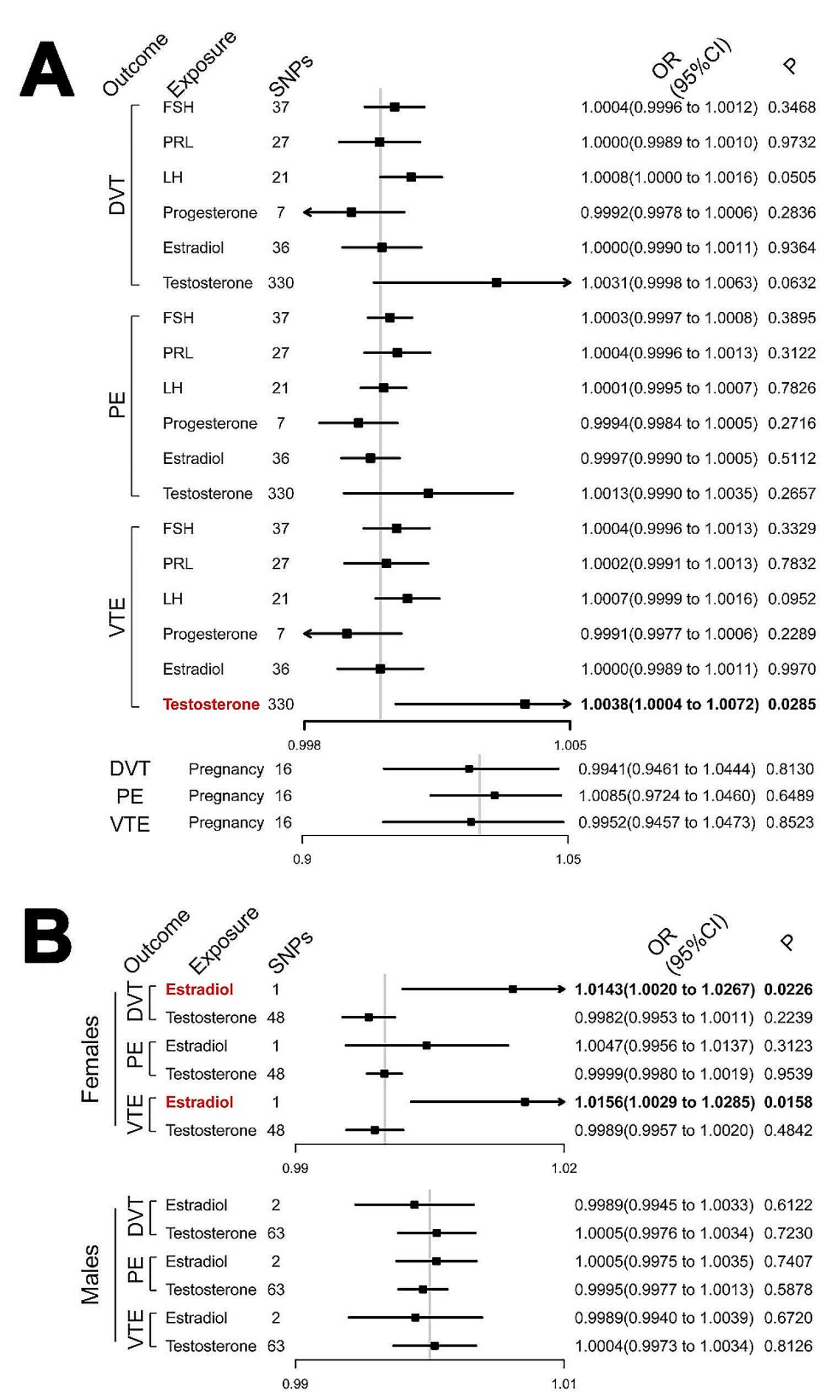
Associations of pregnancy and hormones modulated by the pituitary-gonadal axis with venous thromboembolism (VTE), deep vein thrombosis (DVT), and pulmonary embolism (PE). **A**, General analysis of the total investigated population; **B**, Sex-stratified analysis of estradiol and testosterone. SNPs, single-nucleotide polymorphisms; OR, odds ratio; CI, confidence interval; FSH, follicle-stimulating hormone; PRL, prolactin; LH, luteinizing hormone. Bold values indicate the statistical significance at a level of *P* < 0.05

According to the initial MR analysis of the pituitary-gonadal axis, genetically predicted testosterone concentrations were positively associated with the risk of developing VTE (OR = 1.0038, 95%CI [1.0004–1.0072], *P* = 0.0285), while the influence of genetically predicted estradiol on VTE was not significant (Fig. 4A). In addition, no significant correlation was found between other hormones of the pituitary-gonadal axis (e.g., follicle-stimulating hormone [FSH], prolactin [PRL], luteinizing hormone [LH] or progesterone) and the risk of developing DVT, PE or VTE. Stratification analysis for testosterone and estradiol concentrations adjusted by sex was then performed and revealed that a genetic predisposition to greater estradiol concentrations was associated with a greater risk of developing DVT (OR = 1.0143, 95%CI [1.0020–1.0267], *P* = 0.0226) and VTE (OR = 1.0156, 95%CI [1.0029–1.0285], *P* = 0.0158) in females (Fig. 4B). SHBG rs858518, which is known as a variant associated with sex hormone-binding globulin (SHBG), was identified as the only SNP that was associated with an increased risk of developing VTE, mediated by estradiol concentrations, in females. However, testosterone was no longer directly associated with the risk of developing VTE in either males or females according to the stratification analysis. The MR-PRESSO and Cochrane’s Q tests suggested the presence of horizontal pleiotropy and heterogeneity in the results of sex-stratified testosterone.

A summary of the MR and sensitivity analyses is shown in Supplementary Table 2, indicating consistent results except for the abovementioned outliers. And a list of SNPs which are associated with hormones and hormone-mediated diseases included in this study is presented in Supplementary Table 3.

## Discussion

This MR study demonstrated that genetically predicted hyperthyroidism status and increased FT4 concentrations were associated with a greater risk of VTE. This finding was inconsistent with the results of a previous MR study by Larsson et al. [23], in which no statistically significant associations between genetically predicted thyroid function and VTE were detected. These disparities may be derived from the differences in sources and sample sizes of the data analysed in the two studies. However, another MR study regarding the effects of thyroid function on coagulation and fibrinolysis revealed that genetically predicted hyperthyroidism was associated with increased VWF and FVIII, resulting in a hypercoagulable and hypofibrinolytic state [24]. Genetically predicted increased FT4 concentrations were also associated with increased VWF concentrations [24]. Similarly, a population-based case-control study (the MEGA study) demonstrated that high levels of FT4 increase the concentrations of FVIII, FIX, fibrinogen, and VWF [25]. VWF is secreted by the vascular endothelium and platelets, promoting platelet aggregation and adhesion to the vascular endothelium during vascular injury [26, 27]. Besides, VWF acts as a carrier for FVIII to protect it from degradation [27]. FVIII plays an essential role in hemostasis and has been demonstrated to be a prevalent, dose-dependent risk factor for VTE [28]. These findings indicate a potential mechanism by which thyroid dysfunction is associated with an increased risk of developing VTE via its effects on VWF and FVIII. Our study confirmed that genetically predicted hyperthyroidism and increased FT4 concentrations were important risk factors for VTE, providing more genetic evidence for the causal relationship between thyroid dysfunction and VTE observed in previous observational studies [2, 29–31].

Unexpectedly, this study did not demonstrate any genetic associations between VTE and the hormones regulated through the pituitary-adrenal axis (i.e., ACTH, cortisol, and aldosterone) or related diseases (e.g., Cushing’s syndrome and hyperaldosteronism). Although increased risks of several cardiovascular events were observed in hyperaldosteronism patients, the relationship between hyperaldosteronism and VTE has not been systematically identified [4, 5]. Several previous observational studies have indicated that Cushing’s syndrome is associated with a high risk of developing VTE [7, 8, 32]. The use of exogenous glucocorticoids was identified as a risk factor for VTE [33]. However, some debates remain on whether the relationship between exogenous glucocorticoid use and the risk of developing VTE is causal or is influenced by the underlying disease for which it is used (e.g., inflammatory disease) [4, 5]. The interference of environmental factors, such as interactions between genes and the environment, may have led to the inconsistent results between original observational studies and MR studies [18]. Interestingly, a recent MR study by Allara et al. [34] revealed that higher genetically predicted cortisol levels were related to a decreased risk of VTE (OR = 0.73, 95%CI [0.62–0.87], *P* < 0.001), which was not consistent with our results. We believe that selection bias in terms of sample data sources between these two studies may have contributed to this inconsistence. The summary statistics of VTE, DVT, and PE obtained from UKB and FinnGen in the present study were not identical with theirs. And they have included the data from the International Network Against Venous Thrombosis (INVENT) consortium, which we could not obtained. However, Allara et al. finally demonstrated that the significant relationship between a greater cortisol level and a decreased risk of VTE did not persist after adjustment for systolic blood pressure (OR = 1.06, 95%CI [0.70–1.61], *P* = 0.780). They considered that the association between cortisol and VTE may be mediated by blood pressure [34]. Although our study did not draw similar conclusions, we provided evidence for the view that cortisol concentrations were not directly related to the risk of developing VTE. However, further prospective clinical trials with higher evidence quality are required to resolve the controversies in existing studies.

Another concern in this study was the causal relationship between sex hormones and VTE. Although estradiol has been widely identified as a risk factor for VTE [9, 10, 35], our initial MR analysis did not reveal the significant influence of genetically predicted estradiol on the risk of VTE, while genetically predicted testosterone concentrations were positively associated with the risk of VTE. Further analysis stratified by sex demonstrated that a higher genetically predicted estradiol increased the risk of developing VTE in females, while the influence of testosterone on VTE did not persist. It seemed that the effects of genetically predicted sex hormone concentrations on the risk of developing VTE in the initial MR analysis were confounded by sex, suggesting that the potential mechanisms by which sex hormones influence the risk of VTE differ between males and females.

A previous MR study by Luo et al. [36], using variants in the *JMJD1C* gene region to predict endogenous testosterone, revealed that increased testosterone concentrations were associated with an increased risk of VTE in men, but the association was not significant in women. Besides, Nethander et al. [37] confirmed that the endogenous testosterone predicted by the *JMJD1C* gene was positively associated with the risk of VTE in men, while the endogenous estradiol genetically predicted by variants in the *CYP19A1* gene region was inversely related to the risk of VTE in men. Variants in the *JMJD1C* gene region were identified to be associated with serum testosterone concentrations, with genome-wide significance [36]. *CYP19A1* variants are considered robust IVs for predicting estradiol concentrations because this gene region encodes the aromatase enzyme, which directly metabolizes testosterone to estradiol [37]. It was then speculated that the rate of testosterone conversion to estradiol may influence the risk of VTE in men [37, 38]. The association between a higher testosterone concentration and an increased risk of VTE in the total investigated population observed in our initial MR analysis may have been driven by the association between testosterone and VTE in males. Although the sex-stratifying analysis did not persist the significant influence of testosterone on VTE. This may be partly attributed to the unreliability of testosterone data in stratification analysis, presenting as horizontal pleiotropy and heterogeneity. Screening more reliable data and further verification are needed to clearly reveal the relationship between testosterone and VTE.

On the other hand, the stratification analysis demonstrated that genetically predicted greater estradiol concentrations were associated with a greater risk of developing VTE in females. The identified SNP, SHBG rs858518, was demonstrated for the first time to play an important role in the causal relationship between estradiol and VTE. SHBG rs858518 is known as a variant associated with a decreased level of sex hormone-binding globulin (SHBG) [39]. It is in the promoter region of the SHBG gene and is suggested to be associated with maximal promoter activity. Besides, SHBG rs858518 is in strong linkage disequilibrium with SHBG rs727428 (lying close to the area of high conservation beyond the end of the SHBG gene and being relevant for the binding of transcription factors). In fact, all the haplotypes clearly associated with lower SHBG levels carry minor alleles at both SHBG rs858518 and SHBG rs727428 ^39^. On the other hand, SHBG is a plasma glycoprotein that can regulate the transport, bioavailability, and metabolism of estradiol through binding with it [40]. Previous studies declared that any genetic variant decreasing the levels of SHBG would allow more free estradiol to circulate and hence could increase the risk of diseases associated with increased free estradiol levels (e.g., breast cancer) [39]. It is therefore speculated that SHBG rs858518 may increase the plasma concentration of free estradiol by decreasing SHBG levels, aggravating the effects of estradiol on increasing VTE risk. Based on this inference, SHBG rs858518 may become a potential target for the prevention and treatment of VTE in females.

To our knowledge, this is the first MR study conducted to comprehensively investigate the causal relationship between the hormones regulated through the pituitary-thyroid/adrenal/gonadal axis and the risk of developing VTE. A strength of this study is the MR approach, which reduces systematic biases (e.g., reverse causality and residual confounding) compared with traditional observational studies. In addition, the genetic analysis is based on large-scale GWAS datasets, providing more precise estimates of causal effects with sufficient statistical strength. A series of MR estimates and sensitivity analyses were performed to verify the stability and reliability of our findings.

However, some limitations should be noted. First, like any other MR studies, we cannot entirely exclude the possibility that unobserved pleiotropy might conceal the relatively weak causal effects or bias the established associations. Second, all datasets we used came from European descent, limiting the applicability of our results to different races. Third, the summary statistical data analysed in this study did not include any detailed demographic characteristics or clinical data, restricting our ability to perform subgroup analyses. The levels and normal ranges of hormones change at different ages. Subgroup analysis adjusted for age could provide more specific and practical results. Finally, some odds ratio values were very close to 1 (e.g., ORs of FT4 on DVT/VTE), which may limit the clinical relevance of these variables. This was likely due to the wide range of hormone changes in large sample populations. However, the narrow 95% confidence intervals increased the precision of these significant causal relationships to some extent.

## Conclusion

In this MR study, we confirmed that genetically predicted hyperthyroidism and increased FT4 concentrations were associated with a greater risk of VTE. Besides, the effects of genetically predicted sex hormone concentrations on the risk of developing VTE differed between males and females. Genetically predicted estradiol increased the risk of VTE in females, while the SHBG rs858518 variant may become a potential target for the prevention and treatment of VTE in females. However, the current results cannot demonstrate a direct causal relationship between sex hormones and VTE in males.

### Electronic supplementary material

Below is the link to the electronic supplementary material.

**Supplementary Table 1** Detailed information regarding the GWAS data sources used in this Mendelian randomization study

**Supplementary Table 2** Summary results of several Mendelian randomization analyses and sensitivity analyses

**Supplementary Table 3** Single-nucleotide polymorphisms selected in this study that were associated with hormones and hormone-mediated diseases modulated by the pituitary-thyroid/adrenal/gonadal axis

## Data Availability

All the research data supporting reported results can be found in publicly available GWASs dataset. The detailed information regarding GWAS data sources of this study is presented in Supplementary Table 1.

## Abbreviations

ACTH: Adrenocorticotropic hormone
DVT: Deep venous thrombosis
FT3: Free triiodothyronine
FT4: Free thyroxine
FSH: Follicle-stimulating hormone
GWASs: Genome-wide association studies
IVW: Inverse variance weighting
IV: Instruments variable
LH: Luteinizing hormone
MR: Mendelian randomization
MR-PRESSO: Mendelian Randomization Pleiotropy RESidual Sum and Outlier
PE: Pulmonary embolism
PRL: Prolactin
SNPs: Single-nucleotide polymorphisms
SHBG: Sex hormone-binding globulin
TSH: Thyroid stimulating hormone
UKB: UK Biobank
VTE: Venous thromboembolism
